# Association of Technology-Related Skills and Self-Efficacy With Willingness to Participate in Heart Failure Telemonitoring: Cross-Sectional Observational Study

**DOI:** 10.2196/68992

**Published:** 2025-06-12

**Authors:** Sharon Cuppen, Mayke van Leunen, Tamara Henken, Mayra Goevaerts, Martijn Scherrenberg, Maarten Falter, Paul Dendale, Hareld Kemps, Willem J Kop

**Affiliations:** 1 Department of Medical Psychology, Máxima Medisch Centrum, Veldhoven, The Netherlands; 2 Department of Medical and Clinical Psychology, Tilburg University, Tilburg, The Netherlands; 3 Department of Industrial Design, Eindhoven University of Technology, Eindhoven, The Netherlands; 4 Department of Cardiology, Máxima Medisch Centrum, De Run 4600, Veldhoven, 5504 DB, The Netherlands, 31 40 - 888 - 8000; 5 Faculty of Medicine and Life Sciences, Hasselt University, Hasselt, Belgium; 6 Department of Cardiology, Jessa Hospital, Hasselt, Belgium

**Keywords:** heart failure, telemonitoring, participating in telemonitoring, technological skills, technological self-efficacy, technological learnability

## Abstract

**Background:**

The adoption of telemonitoring in patients with heart failure (HF) is influenced by technology-related skills and self-efficacy, as well as psychological, clinical, and demographic factors. However, the relative importance of these factors with regard to willingness to use telemonitoring is insufficiently understood.

**Objectives:**

This cross-sectional observational study examines the extent to which technology-related skills and self-efficacy are related to willingness to participate in telemonitoring in patients with HF.

**Methods:**

Patients completed questionnaires during hospitalization. Associations of technological skills and self-efficacy with willingness to participate in telemonitoring (dichotomous and continuous scale) were examined using regression models. Mediation-moderation analyses were used to investigate the role of self-efficacy in the association of technological skills with willingness to participate.

**Results:**

This study recruited 61 patients admitted for decompensated HF (mean age 79.9, SD 9.5 years; 24 women). Higher levels of technological skills were associated with higher willingness to participate in telemonitoring (odds ratio [OR] 1.073 per scale unit, 95% CI 1.031-1.117). Technological self-efficacy and learnability were also related to willingness to participate (OR 1.141, 95% CI 1.039-1.252; OR 1.029, 95% CI 1.006-1.052) but did not mediate the association of technological skills with willingness to participate in telemonitoring. Psychological factors (anxiety, depressive symptoms, and perceived social support), age, and cognitive and physical functioning did not moderate the association of technological skills with willingness to participate in telemonitoring.

**Conclusions:**

Technological skills, self-efficacy, and learnability are interrelated factors that need to be considered in patients with HF who are eligible for telemonitoring. Future intervention studies that target these factors could increase patients’ willingness and competence in using telemonitoring after admission for HF.

## Introduction

### Background

Heart failure (HF) is a global pandemic leading to poor quality of life and high mortality and morbidity rates, affecting approximately 64.3 million people worldwide [1]. As a result of global population growth, ageing, and improved survival after diagnosis, the prevalence of HF is further increasing leading to a considerable and growing burden on health care costs [2-4]. Innovative strategies are needed to counteract the effects of HF as a considerable socioeconomic and medical burden, especially to lower hospitalization as they account for 50% of total costs and lead to further physical and physiological deconditioning [4,5].

### Theoretical Framework

eHealth interventions reduce mortality and readmission rates in patients with HF, while improving their medication adherence and self-care behavior [6]. Telemonitoring is a specific application of eHealth that has the potential to reduce hospitalizations, mortality, and health care costs [7]. Health care professionals use telemonitoring to closely review patient-generated health data to detect early signs of HF progression [7]. As reported in a recent meta-analysis, optimal home telemonitoring reduces HF-related hospitalizations by 15%‐19% and all-cause mortality by 16% [8]. Despite these promising outcomes of telemonitoring, its use is not optimal and requires improvements [9].

Important barriers in using telemonitoring are limited technological skills and low technology-related self-efficacy as well as cognitive and physical limitations that are common in patients with HF [10,11]. Cognitive limitations (eg, memory, concentration, and executive functioning) and physical or functional limitations (eg, impaired vision or hearing and presence of hand tremor) are common in older adults and can interfere with using telemonitoring, since these impairments can affect one’s technological capabilities [10-13]. Lack of computer capabilities and insufficient technology onboard training negatively influence the use of technological interventions [14]. In addition, low digital self-efficacy is reported for patients with HF, which might be related to low learnability of digital skills and decreased acceptance and willingness to participate in telemonitoring programs [10]. Furthermore, higher age, female sex, and lower educational level are associated with lower acceptance of telemonitoring [10,15,16]. Evidence also suggests that older adult women are less likely to adopt digital devices than older adult men [17].

For telemonitoring to succeed, patients need to engage in self-management, which requires self-efficacy. Self-efficacy is based on the Social Cognitive Theory of Bandura and refers to the confidence an individual has in his or her own ability to perform a specific behavior [18]. Prior research has shown that experience with computers and telemonitoring devices positively influences the intention to use technological health applications. This relationship is partly and positively mediated by an individual’s perceived self-efficacy [19]. There is an inverse association between self-efficacy and several psychological factors, particularly anxiety and depression. A considerable proportion of patients with HF have anxiety and depressive symptoms (with recent prevalence estimates of 30% and 21.5%, respectively) [20,21]. These psychological factors are also associated with poor health outcomes and higher rehospitalization and mortality rates [22]. The negative association between anxiety and depression with self-efficacy might interfere with telemonitoring participation and thereby adversely influence health outcomes.

In contrast to the potential adverse effects of anxiety and depressive symptoms, social support can improve a patient’s self-efficacy [23,24]. As HF is more common among older adults and age is directly related to less technological skills [25], it seems plausible that higher levels of social support would enhance a patients’ willingness to participate in telemonitoring, particularly in case of limited technological skills or self-efficacy. This perspective is supported by the finding that low social support is a barrier in effectively using medical technology and medical systems [10]. Self-efficacy is a significant mediator in the relationship between social support and self-management behaviors (including treatment adherence) in individuals with HF [23,24].

### Objectives

Based on this background, this study investigated the association of technological skills with technological self-efficacy and learnability in the context of willingness to participate in telemonitoring among patients with HF. Second, the role of psychological factors (anxiety, depressive symptoms, and social support) and demographic as well as functional factors in these associations was explored.

## Methods

### Design and Participants

This study is a single-center cross-sectional observational study among adults with HF who were admitted to the cardiology department for acute decompensated heart failure (ADHF) at Máxima Medical Centre in Veldhoven, the Netherlands. Participants were enrolled from the cardiology department between January and May 2023. Inclusion criteria were a diagnosis of HF according to the European Society of Cardiology guideline definition [26], age of 18 years and older, admission for ADHF, and proficiency in the Dutch language. Patients with a psychiatric disorder or major cognitive impairment (eg, dementia) were excluded.

In total, 119 eligible patients were asked for study participation of whom 61 (51%) gave informed consent. Reasons for nonparticipation (N=58) were not interested in study participation (44, 76%), expected study participation to be too demanding (5, 9%), difficulties understanding the purpose of the study (3, 5%), or death before completing the questionnaires (6, 10%). A graphical abstract of the study can be found in Multimedia Appendix 1.

### Ethical Considerations

This study was evaluated by the medical ethics review board of Máxima Medical Centre and received expedited review, given the nature of the assessments and demands on the participating patients (protocol no. N23.007). Written informed consent was provided to each consecutive patients before inclusion in the study. The study was conducted in accordance to the Declaration of Helsinki. Data were entered in an electronic database using deidentified code numbers. The participants did not receive any form of financial compensation for participating in this trial.

### Procedure

Consecutive patients admitted to the cardiology department meeting the eligibility criteria were asked for study participation by the research team (physician or second-year medical psychology master student). All patients with ADHF who were interested in completing the questionnaires regardless of actual telemonitoring participation were enrolled after signing the informed consent document. Participants received a brief written background description on telemonitoring explaining that participation includes daily measuring of blood pressure, heart rate, and body weight. Participants were informed that these measures, along with answers on a brief daily questionnaire on HF-related complaints, are forwarded to the health care professional using an online website in order to enable proper monitoring and, therefore, better cardiovascular care. Participants completed the study questionnaires on paper to avoid bias in participation and responses relevant to technological skills. Data received from the questionnaires were entered in an electronic database using deidentified code numbers.

### Measures

The “predictor” measures were technological skills, technological self-efficacy, and learnability. The primary outcome measure was willingness to participate in telemonitoring (assessed dichotomously and continuously). We also documented whether or not patients actually participated in telemonitoring after discharge. Additional predictor measures were psychological factors (anxiety, depressive symptoms, and perceived social support) and background factors (demographics, clinical measures, and cognitive and physical functioning).

#### Technological Skills and Learnability

The Digital Health Readiness Questionnaire (DHRQ; Dutch version) was used to investigate technological skills, digital literacy, and learnability [27]. This self-report questionnaire, developed at Hasselt University, consists of 20 questions rated on a 5-point Likert scale, with 1 indicating “totally disagree” to 5 indicating “totally agree.” The questionnaire includes five subdomains: (1) digital access, (2) use of digital technology, (3) digital literacy, (4) digital health literacy, and (5) digital learnability. Subdomains 1-4 (15 items; total scores ranging from 15 to 75) represent technology-related skills (DHRQ_Skills_), described as “digital readiness” by Scherrenberg et al [27]. Subdomain 5 (5 items; total scores ranging from 5 to 25) represents learnability (DHRQ_Learnability_) [27]. Higher scores indicate higher levels of technological skills or learnability. The psychometric properties of the DHRQ are good with acceptable internal consistency (Cronbach α>0.70 for all subdomains) [27]. In this study, the internal consistency of the DHRQ total score was good (Cronbach α=0.96).

#### Technological Self-Efficacy

The modified Computer Self-Efficacy Scale was used to measure technological self-efficacy. This self-report questionnaire has 10 statements that respondents answer based on a 10-point Likert scale, from 1 referring to “not at all confident” to 10 referring to “completely confident” [28]. Total scores range from 10 to 100, with higher scores indicating higher levels of computer-related self-efficacy. Within patients in a clinical rehabilitation setting, a Cronbach α value of 0.94 has been found, indicating a high internal consistency [28].

#### Willingness to Participate in Telemonitoring

Assessment of willingness to participate in telemonitoring was measured with a dichotomous yes or no question as the primary outcome and a continuous measure as a secondary outcome. In addition to reporting whether or not they would participate in telemonitoring (dichotomous outcome), participants were also asked the following question: “Based on a scale from 1 (not at all) to 10 (very likely), how likely is it that you would participate in telemonitoring if you had sufficient technological skills and/or help from friends or family with using the technology?” (continuous outcome).

#### Psychological Measures

The Generalized Anxiety Disorder-7 (GAD-7) Scale was used to provide information regarding the presence and degree of anxiety symptoms [29]. The Patient Health Questionnaire-9 (PHQ-9) was used to assess the presence and degree of depressive symptoms [30]. Participants were asked to rate symptoms on a 4-point Likert scale, with 0 indicating “not at all” to 3 “nearly every day” for both GAD-7 and PHQ-9. Total scores of answers range from 0 to 21 for the GAD-7 and 0 to 27 for the PHQ-9. Higher scores indicate a higher level of symptoms, with a cutoff score of ≥10 for both scales to indicate levels that are likely to meet diagnostic criteria for anxiety and depression. The GAD-7 and PHQ-9 are recommended for psychological assessment of cardiovascular outpatients [31]. Research has demonstrated good internal consistency (Cronbach α=0.82 and 0.83, respectively) [22,32,33].

The Multidimensional Scale of Perceived Social Support (MSPSS) was used to measure perceived social support. This questionnaire consists of 12 items with 4 items representing each of the 3 subdomains: support from family, friends, or significant others, respectively [34,35]. Questions are based on a 7-point Likert scale, from 1 “very strongly disagree” to 7 “very strongly agree.” Total scores range from 12 to 84, with higher scores indicating more perceived social support. The MSPSS has good psychometric properties with a Cronbach α=0.94 for the total instrument [34].

#### Background Variables

Sociodemographic characteristics regarding age, sex, and educational level were obtained with a self-report questionnaire designed for the purposes of this study. Clinical information relevant to HF was obtained from the medical record, including left ventricular ejection fraction (LVEF).

The self-report Cognitive Failure Questionnaire (CFQ) was used to provide information regarding cognitive functioning, with 25 items measuring the frequency of everyday cognitive mistakes [36,37]. Items are rated on a 5-point Likert scale, with 0 indicating “never” to 4 indicating “very often.” Total scores range from 0 to 100, with higher scores indicating a higher level of cognitive problems. The CFQ has good psychometric properties within the general adult population (Cronbach α=0.89) [34].

The Computer-related Physical Functioning Questionnaire, developed for the purposes of this study, was used to obtain information about 3 domains of physical functioning: vision, hearing, and presence of hand tremors. This self-report inventory includes 6 items, 2 items for each domain measured. Items are rated on a 4-point Likert scale, with 0 indicating “not at all” to 3 indicating “almost all the time” (eg, “I experience problems with my eyesight,” and “I am limited in using the computer because of motor problems in my hands.”). In this sample, the Cronbach α value was 0.51. Total scores can range from 0 to 18, with higher scores indicating more physical limitations.

### Statistical Analyses

Bivariate associations between the variables investigated in this study were examined using Pearson correlations and independent sample 2-tailed *t* tests. To investigate the association of “technological skills” with willingness to participate in telemonitoring (dichotomous outcome), logistic regression analyses were used. Multiple logistic regression analysis was used to adjust for technological self-efficacy and learnability, as well as for demographic (age, sex, and education level) and function-related (cognitive functioning and physical limitations) variables. Results for the continuous outcome of willingness to participate in telemonitoring were analyzed using (multiple) linear regression models and presented as standardized regression coefficients (β). The overall fit of the linear regression models was examined using the total *R*^2^. Participants with missing values were excluded from analyses using a pairwise approach for bivariate models and listwise for multivariable models. Relevant assumptions for logistic and linear regression were evaluated and met prior to conducting the analyses.

To investigate whether technological self-efficacy and learnability played a mediating role in the association between technological skills and willingness to participate in telemonitoring, the PROCESS tool of the Statistical Package for the Social Sciences (SPSS) was used. The overall fit was examined using Nagelkerke *R*^2^ for analyses with the dichotomous outcome variable and *R*^2^ for analyses with the continuous outcome variable. Associations were evaluated using a type I error (2-sided α value) of .05 or a 95% CI. All statistical analyses were conducted using the SPSS software (version 28; IBM Corp).

### Statistical Power and Sample Size

The sample size of this study was based on medium to large effect sizes that are likely to be clinically relevant, as no prior research has investigated the research question addressed in this study. The sample of 61 participants enables detection of a bivariate correlation of 0.35 and an OR of 2.2 (assuming that 50% of the participants will have high levels of technological skills), with a power of 0.80 and a 2-sided α value of .05.

## Results

### Participant Characteristics

The mean age of the sample (N=61) was 79.9 (SD 9.5) years (range: 49-96 years) and 37 (61%) were male (Table 1). Approximately 48% (29/61) had completed an education beyond high school. The mean LVEF was 40 (SD 16%), with 24 patients (39%) being classified as HF with reduced ejection fraction. Four participants (7%) had already participated in telemonitoring prior to study participation.

**Table 1.** Descriptive statistics of patients with heart failure during hospital admission (N=61).

Characteristics	Values
Demographic characteristics	
Age (years), mean (SD)	79.9 (9.5)
Sex, male, n (%)	37 (61)
Education, n (%)	
Elementary or high school	31 (51)
Secondary vocational education	14 (23)
Higher vocational education	9 (15)
College or university education	6 (10)
Clinical characteristics	
LVEF^a^, mean (SD)	40.3 (15.6)
HF^b^ classification, n (%)	
LVEF ≥50%	28 (46)
LVEF 41%‐49%	8 (13)
LVEF ≤40%	24 (39)
BMI (kg/m^2^), at discharge, mean (SD)	25.7 (4.9)
History of cerebrovascular accident, n (%)	11 (18)
History of myocardial infarction, n (%)	12 (19)
Percutaneous coronary intervention, n (%)	16 (25)
Coronary artery bypass grafting, n (%)	3 (5)
Peripheral artery disease, n (%)	14 (22)
Arterial fibrillation, n (%)	31 (48)
Diabetes mellitus, n (%)	17 (26)
Hypertension, n (%)	37 (57)
Hypercholesterolemia, n (%)	13 (20)
Psychological and technology-related measure	
Cognitive functioning (CFQ^c^), mean (SD)	28.8 (17.7)
Physical limitations (CPFQ^d^), mean (SD)	4.5 (3.3)
Anxiety (GAD-7^e^), mean (SD)	5.9 (5.1)
Depressive symptoms (PHQ-9^f^), mean (SD)	8.7 (5.6)
Social support (MSPSS^g^), mean (SD)	67.1 (14.6)
Technological skills (DHRQ^h^_Skills_), mean (SD)	38.4 (17.7)
Technological learnability (DHRQ_Learnability_), mean (SD)	14.4 (6.8)
Technological self-efficacy (mCSES^i^), mean (SD)	53.1 (26.4)
Willingness to participate in TM^j^, yes, n (%)	40 (66)
Willingness to participate in TM with sufficient technological skills or help, mean (SD)	7.1 (2.5)
Started with TM after hospital discharge, n (%)	26 (44)

a LVEF: left ventricular ejection fraction.

b HF: heart failure.

c CFQ: Cognitive Failure Questionnaire.

d CPFQ: Computer-related Physical Functioning Questionnaire.

e GAD-7: Generalized Anxiety Disorder-7.

f PHQ-9: Patient Health Questionnaire-9.

g MSPSS: Multidimensional Scale of Perceived Social Support.

h DHRQ: Digital Health Readiness Questionnaire.

i mCSES: modified Computer Self-Efficacy Scale.

j TM: telemonitoring.

Physical and cognitive functioning were within the normative range, mean Computer-related Physical Functioning Questionnaire 4.5 (SD 3.3) and mean CFQ 28.8 (SD 17.7). Anxiety and depression were above clinical cutoff values (score ≥10) for 14 (23%) and 23 (38%) participants, respectively.

### Measures and Statistical Analyses

#### Willingness to Participate in Telemonitoring

In total, 66% (40/61) of participants were willing to participate in telemonitoring. Patients who affirmed willingness to participate in telemonitoring (N=40) also scored higher on the continuous measure of willingness to participate than those who were not willing to participate (mean 7.9, SD 1.9 vs mean 5.5, SD 2.9; *P*=.002), indicating that both indices assessed the same outcome measure.

Of those 40, a total of 5 were not eligible for telemonitoring after discharge (1 patient moved to another region, 1 died before hospital discharge, and 3 were discharged to a hospice). Of the remaining 35 patients who mentioned wanting to participate in telemonitoring, 24 (69%) actually started with telemonitoring at discharge. In addition, another 10% (2/21) of patients who initially stated that they were not interested in telemonitoring ended up starting with telemonitoring at discharge (total actual participation was 26/61, 43% of the total sample).

#### Technological Skills and Willingness to Participate in Telemonitoring (Unadjusted Analyses)

Higher levels of technological skills were associated with a higher likelihood of willingness to participate in telemonitoring (OR 1.073, 95% CI 1.031-1.117, per DHRQ_Skills_ scale unit) (Table 2). Patients with an above-mean score (≥39) on the DHRQ_Skills_ (32/61, 53%) were approximately 10 times more likely to be willing to participate in telemonitoring than patients with scores below the mean (OR 9.917, 95% CI 2.752-35.740).

**Table 2.** Logistic regression analyses on willingness to participate in telemonitoring in patients with heart failure measured dichotomously during hospital admission.

Variable	Unadjusted analysis			Adjusted analysis^a^		
	OR^b^	95% CI	*P* value	OR	95% CI	*P* value
Technological skills	1.073	1.031-1.117	.001	1.121	1.035-1.215	.01
Age (years)	0.910	0.843-0.983	.02	0.907	0.808-1.018	.10
Sex (0=male; 1=female)	1.083	0.366-3.204	.89	1.084	0.184-6.396	.93
Education level^c^	1.110	0.663-1.858	.69	2.878	1.091-7.591	.03
Physical functioning	0.932	0.788-1.103	.41	1.074	0.819-1.409	.61
Cognitive functioning	1.013	0.981-1.046	.43	0.998	0.947-1.053	.95
Technological learnability	1.141	1.039-1.252	.01	1.171	0.907-1.512	.23
Technological self-efficacy	1.029	1.006-1.052	.01	0.952	0.884-1.024	.19

a Variables are adjusted for each other.

b OR: odds ratio.

c Ranging from college or university education to elementary or high school.

Patients who had higher scores on technological self-efficacy were more likely to be willing to participate in telemonitoring (OR 1.029, 95% CI 1.006-1.052, per modified Computer Self-Efficacy Scale unit). In addition, younger age (OR 0.910, 95% CI 0.843-0.983, per year), more social support (OR 1.045, 95% CI 1.003-1.089, per MSPSS scale unit), and technological learnability (OR 1.141, 95% CI 1.039-1.252, per DHRQ_Learnability_ scale unit) were associated with patients affirming willingness to participate in telemonitoring.

Multivariate analyses (Table 2) showed that the association between technological skills and willingness to participate remained significant when adjusting for age, sex, education level, cognitive functioning, physical limitations, technological learnability, and technological self-efficacy (OR 1.121, 95% CI 1.035-1.215). Additionally, higher education was indicated as a significant predictor in the multivariate model (Table 2, right columns).

When repeating the analyses using willingness to participate as a continuous outcome variable, a similar pattern of results was found. Higher levels of technological skills were associated with higher levels of the continuous measure of willingness to participate (unadjusted *r*=0.384, *P*=.003). The bivariate correlations between willingness to participate (measured as continuous variable) with other variables are displayed in Table S1 in Multimedia Appendix 2. In addition to technological skills, technological self-efficacy (*r*=0.307; *P*=.018), social support (*r*=0.507; *P*<.001), and technological learnability (*r*=0.433; *P*=.001) were associated with the continuous measure of willingness to participate. Older age was correlated with lower levels of technological skills (*r*=−0.465; *P*<.001), lower technological self-efficacy (*r*=−0.484; *P*<.001), and less learnability (*r*=−0.334; *P=*.009) (Table S1 in Multimedia Appendix 2). The association of technological skills with the continuous measure of willingness to participate remained significant when adjusting for covariates (β=.392; *P*=.044, overall model *R*^2^=0.326) (see Table S2 in Multimedia Appendix 2 for details of the multiple linear regression analysis).

#### Mediating Role of Technological Self-Efficacy and Learnability in Willingness to Participate in Telemonitoring

Higher levels of technological skills were strongly and positively correlated with higher levels of technological self-efficacy (*r*=0.74; *P*<.001) and learnability (*r*=0.69; *P*<.001) (Table S1 in Multimedia Appendix 2), 2 constructs that were also both associated to willingness to participate (OR 1.029, 95% CI 1.006-1.052; OR 1.141, 95% CI 1.039-1.252). Table 2 and Table S2 in Multimedia Appendix 2 show that adjusting for these variables did not result in an attenuation of the association between technological skills with willingness to participate in telemonitoring.

To further explore the interplay of pathways involved in participating in telemonitoring, mediation analyses were conducted. These analyses indicated that the positive association between technological skills and willingness to participate was not mediated by technological self-efficacy (*B*=−0.006, 95% CI −0.078 to 0.035) or learnability (*B*=0.008, 95% CI −0.035 to 0.047]) (Table 3, left part). Results for the continuous measure of participation also revealed no mediation of technological self-efficacy (*B*=0.007, 95% CI −0.028 to 0.049) and learnability (*B*=0.031, 95% CI −0.001 to 0.065) in the association between technological skills and willingness to participate in telemonitoring (Table 3, right part).

**Table 3.** The mediating role of technological self-efficacy and learnability, and the moderating role of anxiety, depressive symptoms, and social support in willingness to participate in telemonitoring in patients with heart failure during hospital admission^a^.

	Willingness to participate							
	Dichotomous				Continuous			
	*B* ^b^	95% CI	*P* value	Nagelkerke *R*²^c^	*B*	95% CI	*P* value	*R*²
Mediation effects								
Direct effect of TS^d^ on Y^e^	0.076	0.021 to 0.131	.01		0.049	−0.002 to 0.101	.06	
Indirect effect of TS on Y (mediation of technological SE^f^)	−0.006	−0.078 to 0.035			0.007	−0.028 to 0.049		
Total model			<.001	.316			.01	.150
Direct effect of TS on Y	0.063	0.013 to 0.113	.01		0.025	−0.022 to 0.072	.29	
Indirect effect of TS on Y (mediation of learnability)	0.008	−0.035 to 0.047			0.031	−0.001 to 0.065		
							
Total model			<.001	.319			.002	.203
Moderation effects								
TS	0.037	−0.036 to 0.110	.32		0.089	0.020 to 0.157	.01	
Depression	−0.112	−0.365 to 0.141	.39		0.212	−0.055 to 0.479	.12	
TS^g^ depression			.35				.35	
Total model			.002	.314			.01	.427
TS	0.081	0.014 to 0.148	.02		0.050	−0.006 to 0.106	.08	
Anxiety	0.062	−0.206 to 0.330	.65		−0.061	−0.344 to 0.222	.67	
TS^g^ anxiety			.76				.93	
Total model			.001	.319			.02	.396
TS	−0.089	−0.275 to 0.097	.35		0.058	−0.088 to 0.203	.43	
Social support	−0.030	−0.118 to 0.058	.50		0.084	0.009 to 0.158	.03	
TS^g^ Social support			.10				.82	
Total model			<.001	.409			<.001	.580

a Moderation models were conducted for each of the 3 psychological variables separately.

b Unstandardized coefficients.

c Residuals squared.

d TS: technological skills.

e Y: willingness to participate in telemonitoring.

f SE: self-efficacy.

g Interaction effect.

#### Moderating Role of Anxiety, Depressive Symptoms, and Social Support in Willingness to Participate in Telemonitoring

Table 3 (bottom part) indicates that there was no significant moderation (ie, interaction effect) of the variables anxiety (*P*=.76; *P*=.93), depression (*P*=.35; *P*=.35), and social support (*P*=.10; *P*=.82) with technological skills on the dichotomous and continuous outcome measures of willingness to participate in telemonitoring. There were also no significant main effects of these psychological factors, except for social support on the continuous outcome measure (Table 3, bottom part).

## Discussion

### Summary of the Main Findings

This real-life cohort study shows that the level of technological skills is a significant, independent factor in a patient’s willingness to engage in telemonitoring for HF. This association remained significant when taking covariates and moderating factors into account. In addition to technological skills, technological self-efficacy and learnability were found to be important factors in willingness to participate in telemonitoring. Higher technological self-efficacy and learnability levels were associated with both technological skills and willingness to participate in telemonitoring but did not mediate the link between technological skills and willingness to participate. These findings indicate that improving technological skills might help patients with HF to actively adopt remote monitoring options for their clinical care. However, self-efficacy and learnability are additional important target constructs as well since they are strongly and positively associated with technological skills. This study sets the stage for future investigations in which technological self-efficacy and other factors associated with technological skills are addressed with the long-term goal to optimize patient participation in telemonitoring and other forms of telemedicine.

Consistent with previous research, this study indicates that a lack of technological skills is a barrier in participating in telemonitoring among patients with HF [10,11,14,38]. Patients with high technological skills were approximately 10 times more likely to be willing to participate in telemonitoring than patients with low technological skills. These findings suggest that technological skills, self-efficacy, and learnability are strongly and positively intercorrelated, and that these 3 constructs are positively associated with willingness to participate in telemonitoring. In contrast to previous studies, no significant associations were found between age and cognitive dysfunction with willingness to participate in telemonitoring. These discrepancies require further investigations with larger and more heterogeneous samples in terms of education and cultural background.

This study also confirms that technological self-efficacy and learnability play a role in the adoption of health technology [19], but these factors did not mediate the relationship between technological skills and willingness to participate in telemonitoring. This finding might be explained by the high correlation of technological skills with both technological self-efficacy and learnability. Specifically, if one (primary) predictor variable is strongly associated with an outcome measure (in this case technological skills with willingness to participate in telemonitoring), then there is little additional variance left to be explained by other predictors, particularly when these other factors are also highly correlated with the primary predictor. It is also possible that technological self-efficacy and learnability are primary factors in willingness to participate in telemonitoring and that this association is mediated by technological skills, which is the reverse pathway as was investigated in this study.

### Limitations and Strengths

The study has limitations that need to be considered when interpreting the findings. Self-report questionnaires are potentially influenced by socially desirable answers and by persons’ mental state at the time of completion [39]. This study found that approximately two-thirds of participants who mentioned to be willing to participate actually started telemonitoring. It is not known whether this discrepancy reflects patient, health care, or program-related factors (eg, access to the technology, preenrollment support, or system requirements). Also, exclusively Dutch-speaking participants were included, and therefore results cannot be generalized to other (non-Western) cultures [40]. In addition, given the diverse applications of telemonitoring across various levels, our findings are specific to the methodology used in this sample and cannot be extrapolated to all telemonitoring practices (eg, noninvasive vs invasive). A minimal selection bias, moreover, could not be avoided despite mentioning that study participation did not affect the actual participation. Participations with certain characteristics may be more open to participate in research. As a consequence of the limited sample size and the complex statistical models used in this study, exclusively medium to large effect sizes could be detected with sufficient statistical power. Among the strengths of the study are the assessment of a wide range of relevant covariates, including cognitive functioning, and the fact that the sample of the study is comparable with the typical HF population (eg, high age and representative LVEF groups) [41,42].

### Recommendations for Future Research

Future longitudinal and intervention research is needed to better understand the association of technological skills, self-efficacy, and learnability with willingness to participate in telemonitoring and to obtain more robust and generalizable results. Investigations using larger samples and a broader range of participants in terms of demographic characteristics are needed to increase generalizability of the present study findings. It would also be useful to include “privacy concerns” in future analyses, as this factor might influence the use and willingness to participate in telemonitoring [40,43]. Additionally, future studies are needed to further examine the gap between willingness to participate and actually participating (ie, the intention-behavior gap) and factors influencing the participation duration [44]. The intention-behavior gap in this study was as follows: two-thirds of the patients expressing willingness actually participated and one-third did not, and approximately one-tenth of the patients who initially expressed no willingness ended up participating in telemonitoring. These numbers are consistent with previous research [45]. Future longitudinal studies are also needed to further examine the optimal time sequence of the intervention targeting technological skills, self-efficacy, and learnability on telemonitoring participation. A step-wise approach could potentially be an effective method to train patients in using telemonitoring devices (eg, starting with measuring weight and gradually expanding tasks).

### Conclusions

This study shows that higher levels of technological skills, self-efficacy, and learnability are associated with a higher likelihood of willingness to participate in telemonitoring among patients with HF. Therefore, clinical practice can be improved by screening for the degree of technological skills (eg, with the DHRQ or other assessment tools) to identify patients who need onboard training and to further increase participation and efficacy of telemonitoring. It will be important to reduce the gap between willingness to participate and actual participation, and to develop interventions targeting higher adoption of HF telemonitoring.

## Supplementary material

10.2196/68992 Multimedia Appendix 1 Graphical abstract.

10.2196/68992 Multimedia Appendix 2 Supplementary information on the correlations observed with willingness to participate and other variables, as well as the relationship between technological skills and willingness to participate in telemonitoring.

## Abbreviations

ADHF: acute decompensated heart failure
CFQ: Cognitive Failure Questionnaire
DHRQ: Digital Health Readiness Questionnaire
GAD-7: Generalized Anxiety Disorder-7
HF: heart failure
LVEF: left ventricular ejection fraction
MREC: Medical Research Ethics Committee
MSPSS: Multidimensional Scale of Perceived Social Support
PHQ-9: Patient Health Questionnaire-9
