# Supplementary figures and images for: Association of Technology-Related Skills and Self-Efficacy With Willingness to Participate in Heart Failure Telemonitoring: Cross-Sectional Observational Study
Source: JMIR Form Res. 2025 Jun 12;9:e68992. doi: 10.2196/68992 (PMC12178583; doi:10.2196/68992)

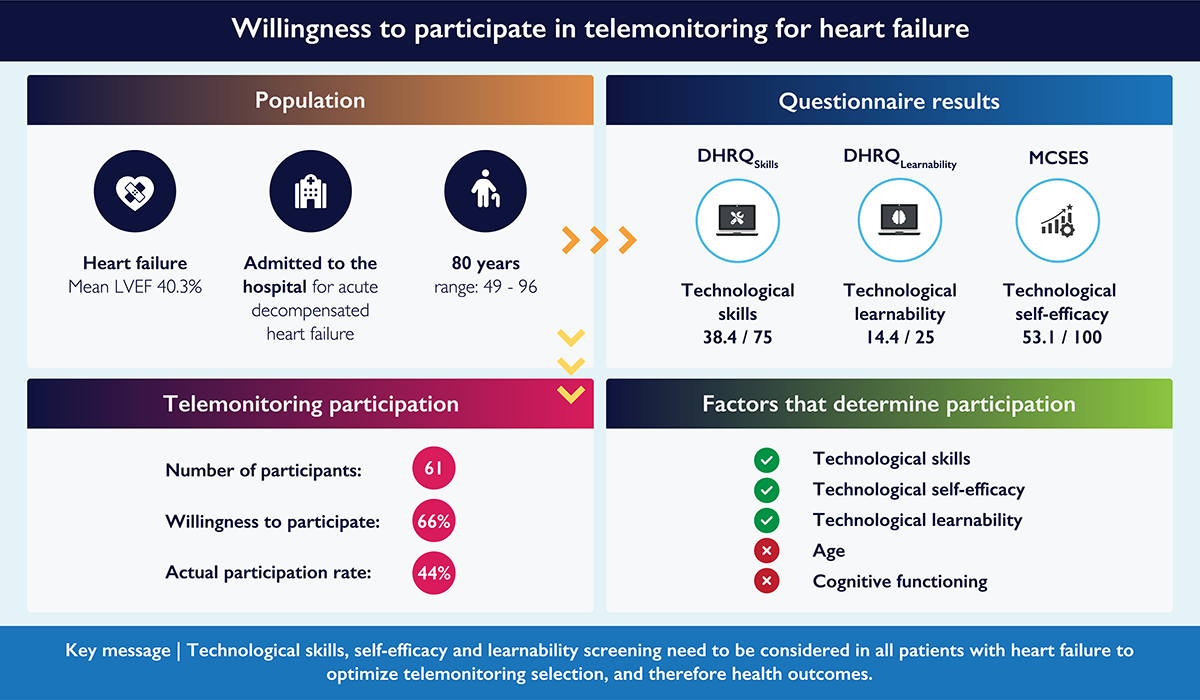

Supplement: Multimedia Appendix 1 [file formative-v9-e68992-s001.png]
