# Supplementary material for: Association of Technology-Related Skills and Self-Efficacy With Willingness to Participate in Heart Failure Telemonitoring: Cross-Sectional Observational Study
Source: JMIR Form Res. 2025 Jun 12;9:e68992. doi: 10.2196/68992 (PMC12178583; doi:10.2196/68992)
Supplement: Multimedia Appendix 2 [file formative-v9-e68992-s002.docx]

**Supplementary tables**

**Table S1.** Correlations of dependent variables, independent variables, and covariates/moderators in patients with heart failure during hospital admission.

|  | |  | 1 | 2 | 3 | 4 | 5 | 6 | 7 | 8 | 9 |
| --- | --- | --- | --- | --- | --- | --- | --- | --- | --- | --- | --- |
|  | |  |  |  |  |  |  |  |  |  |  |
| 1. Participation (cont)^b^ | |  |  |  |  |  |  |  |  |  |  |
|  | *r* |  | 1 | .384 | .433 | .307 | -.162 | .119 | -.065 | -.076 | -.188 |
|  | *P* value |  |  | **.003** | **.001** | **.02** | .22 | .37 | .63 | .57 | .16 |
| 2. Technological skills | |  |  |  |  |  |  |  |  |  |  |
|  | *r* |  |  | 1 | .692 | .737 | -.465 | -.221 | .433 | .156 | -.373 |
|  | *P* value |  |  |  | **< .001** | **< .001** | .088 | **.001** | **.001** | .23 | **.004** |
| 3. Technological learnability | |  |  |  |  |  |  |  |  |  |  |
|  | *r* |  |  |  | 1 | .836 | -.334 | -.099 | .266 | .117 | -.313 |
|  | *P* value |  |  |  |  | **< .001** | **.01** | .45 | **.04** | .37 | **.02** |
| 4. Technological SE^a^ | |  |  |  |  |  |  |  |  |  |  |
|  | *r* |  |  |  |  | 1 | -.484 | -.162 | .308 | .214 | -.220 |
|  | *P* value |  |  |  |  |  | **< .001** | .21 | **.02** | .10 | .10 |
| 5. Age | |  |  |  |  |  |  |  |  |  |  |
|  | *r* |  |  |  |  |  | 1 | .183 | -.198 | -.105 | .148 |
|  | *P* value |  |  |  |  |  |  | .16 | .13 | .42 | .27 |
| 6. Sex^c^ | |  |  |  |  |  |  |  |  |  |  |
|  | *r* |  |  |  |  |  |  | 1 | -.376 | -.160 | .132 |
|  | *P* value |  |  |  |  |  |  |  | **.003** | .22 | .32 |
| 7. Education level | |  |  |  |  |  |  |  |  |  |  |
|  | *r* |  |  |  |  |  |  |  | 1 | -.037 | -.280 |
|  | *P* value |  |  |  |  |  |  |  |  | .78 | **.04** |
| 8. Cognitive functioning | |  |  |  |  |  |  |  |  |  |  |
|  | *r* |  |  |  |  |  |  |  |  | 1 | .200 |
|  | *P* value |  |  |  |  |  |  |  |  |  | .13 |
| 9. Physical limitations | |  |  |  |  |  |  |  |  |  |  |
|  | *r* |  |  |  |  |  |  |  |  |  | 1 |
|  | *P* value |  |  |  |  |  |  |  |  |  |  |

^a^SE: self-efficacy.

^b^Cont: continuous measured.

^c^Sex: male = 0 and female = 1, with negative correlations indicating higher participation rate among men.

**Table S2.** Willingness to participate in telemonitoring given sufficient skills/help in patients with heart failure measured continuously during hospital admission.

|  | Total *R² ^a^* | B^b^ | 95% CI^c^ | |  | *β^d^* | *P^e^* |
| --- | --- | --- | --- | --- | --- | --- | --- |
| Unadjusted *(block 1)* | .148 | .056 | .020 | .092 |  | .384 | **.003** |
| Adjusted for age, sex, education level, cognitive  functioning, and physical limitations *(block 2)* | .256 | .078 | .031 | .124 |  | .522 | **.002** |
| Adjusted for technological self-efficacy and  learnability *(block 3)* | .326 | .058 | .002 | .115 |  | .392 | **.04** |

*^a^R²*: residuals squared.

^b^B: unstandardized coefficients.

^c^CI: confidence interval.

^d^*β*: standardized coefficients.

^e^*P*: *P*-value.
